# Integrin-Mediated Delivery of Drugs and Nucleic Acids for Anti-Angiogenic Cancer Therapy: Current Landscape and Remaining Challenges

**DOI:** 10.3390/bioengineering5040076

**Published:** 2018-09-20

**Authors:** Poulami Majumder

**Affiliations:** 1 Division of Lipid Science and Technology, CSIR-Indian Institute of Chemical Technology, Uppal Road, Tarnaka, Hyderabad 500007, India; poulami.majumder@nih.gov or poulami.orgchem@gmail.com; 2 Chemical Biology Laboratory, National Cancer Institute, 376 Boyles St, Frederick, MD 21702, USA

**Keywords:** Integrin, drug delivery, angiogenesis, anti-angiogenic therapy, siRNA, tumor-homing peptides, combination delivery of drug and nucleic acids

## Abstract

Angiogenesis, sprouting of new blood vessels from pre-existing vasculatures, plays a critical role in regulating tumor growth. Binding interactions between integrin, a heterodimeric transmembrane glycoprotein receptor, and its extracellular matrix (ECM) protein ligands govern the angiogenic potential of tumor endothelial cells. Integrin receptors are attractive targets in cancer therapy due to their overexpression on tumor endothelial cells, but not on quiescent blood vessels. These receptors are finding increasing applications in anti-angiogenic therapy via targeted delivery of chemotherapeutic drugs and nucleic acids to tumor vasculatures. The current article attempts to provide a retrospective account of the past developments, highlight important contemporary contributions and unresolved set-backs of this emerging field.

## 1. Introduction

Drug discovery research during the past several decades has indubitably produced numerous potent anti-cancer therapeutics. A serious set-back associated with the use of many of these drugs is their dose-limiting toxicity. For instance, block-buster chemotherapeutic Doxorubicin is known to induce deleterious cardiotoxic effects. Besides promising initial response post-chemotherapy, tumors often develop drug resistance which reduces penetration of subsequent doses into tumor cells, eventually leading to treatment failure. Non-specific tissue distribution profiles of traditional chemotherapeutics often affect both normal and tumor cells, with only suboptimal dose being able to accumulate into the sites of interest [1]. Improvement of drug efficacy is possible with slow-release technologies which allow the drugs to be delivered at a desired rate over an extended period. Targeted chemotherapy which relies on specifically affecting distinct organs of interest with minimal side effects to the healthy tissues provides an attractive strategy to increase therapeutic index of a drug. Targeting could be passive where nanoparticulate drug accumulates via the leaky vasculature at the tumor site. In contrast, active targeting is mediated through ligand-receptor interaction in the target cells after the nanoparticles reach the site of interest through systemic circulation. Expression of distinct receptors on the target cells relative to non-target cells, internalization capacity of the receptor upon binding to the nanoparticles and density of the receptors on the cell-surface for sufficient interaction with the ligand-decorated particles are the important parameters for successful targeted therapy [2]. Anti-angiogenic therapy, aimed at exploiting unique molecular markers expressed on tumor blood vessels, is one of the most widely explored targeted therapy till date [3]. Angiogenesis, sprouting of new blood vessels from pre-existing vasculatures, is a remarkable feature of tumor growth [4]. Growing tumors get their oxygen and nutrients from these tumor neovasculature (newly formed blood vessels around tumor). Inhibition of angiogenesis, as envisaged, would shut down supply of nutrients to tumor cells and in consequence, cells will die simply out of starvation. This is in contrast to therapeutic angiogenesis which is desired for treating cardiovascular diseases like myocardial infarction [5]. Effective targeting of endothelial cells in the tumor vasculature could be accomplished by utilizing specific receptors over expressed on the surface of tumor endothelial cells, without affecting healthy resting vasculatures. To this end, integrin receptors, the αβ-heterodimeric transmembrane glycoprotein receptors are one of the most highly exploited molecular markers for targeted drug/gene delivery to tumor vasculatures [6,7,8,9,10].

**Figure 1:**
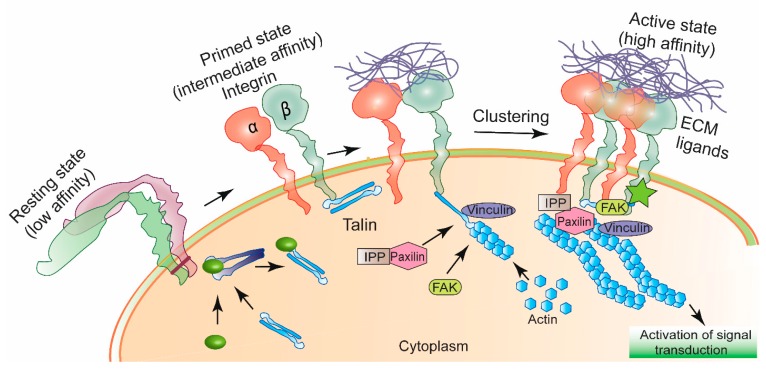
Schematic diagram of integrin activation. Integrins receptors when not in interaction with ligands from extracellular matrix (ECM) stay in an inactive conformation. Specific intracellular protein-induced conformational change in integrins allow them to bind certain ECM ligands. Ligand-bound integrins undergo clustering, gets attached to the actin cytoskeleton via a multiprotein complex of Paxilin, FAK (Focal Adhesion Kinase), IPP (ILK-parvin-PINCH) and Vinculin. Activated integrin participates to control signal transduction for maintaining cellular motility, proliferation and invasion [11].

**Figure 2:**
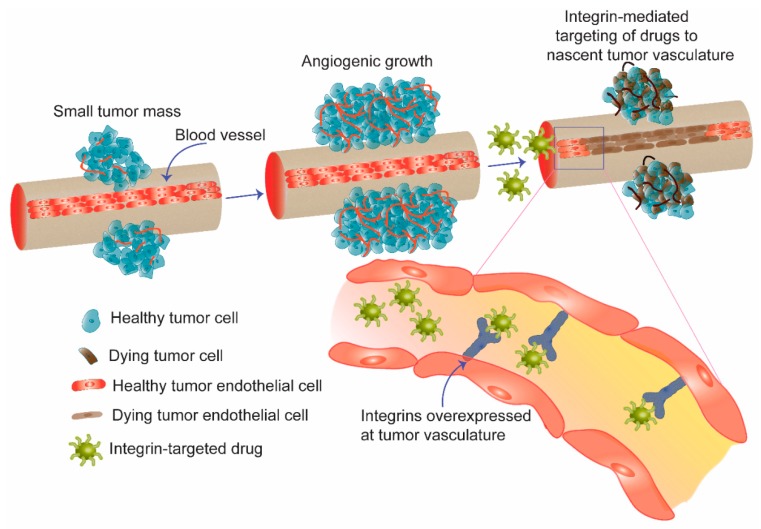
Schematic diagram showing angiogenesis, sprouting of new blood vessels from pre-existing vasculature as an effective mean for tumor growth. In anti-angiogenic therapy, integrin receptors overexpressed on the nascent blood vessels are targeted to deliver drugs and genes selectively to tumor vasculature. Apoptosis of tumor endothelial cells in the vasculature shuts down supply of nutrients to tumor cells and eventually induces killing of bulk tumor.

Integrins play important roles in connecting or integrating the cell cytoskeleton to the extracellular matrix in addition to maintaining cell-to-cell communications [11] (Figure 1). These receptors possess three different structural domains: a large extracellular domain, one transmembrane region and a cytoplasmic region. While the extracellular region controls ligand-binding, the cytoplasmic region is involved in maintaining cellular proliferation, migration, and invasion [12]. In mammals at least 24 different integrins have been identified which result from a combination of α- and β- subunits in a non-covalent fashion [13]. Ever since Cheresh and Coworkers disclosed their findings that αvβ3 integrin receptors are highly upregulated on tumor endothelial cells compared to their expression levels in quiescent vessels of normal tissues [14,15], global efforts are being directed toward designing efficacious systems for selective delivery of cytotoxic agents and nucleic acids to tumor vasculatures via integrin receptors [16,17] (Figure 2). The current review briefly describes recent developments in this emerging field with special focus on integrin-targeted nanoparticles.

## 2. High-Affinity Ligands of Integrin Receptors

In a pioneering study conducted more than 25 years ago, Ruoslahti group demonstrated that the arginine-glycine-aspartic acid (RGD) sequence plays a key role in cellular adhesion of extracellular fibronectin [18]. Subsequently, such RGD-sequences were found to be present in many other extracellular matrices (ECMs) and integrins were identified as their receptors [19,20,21]. Interestingly, many viruses have been found to exploit RGD motif of their surface glycoproteins to potentiate integrin receptor-mediated cellular internalization processes [22]. Stated differently, tripeptide RGD is one of the most evolutionarily conserved and efficient integrin-binding ligands.

An elegant strategy for identifying high-affinity integrin receptor ligands is using phage (a virus that attacks bacteria) display libraries. Selection of engineered phage (from a library of peptides expressed as fusions to phage surface proteins that specifically home to tumor vasculatures upon injection into mice) has provided several potent peptide ligands for αvβ3, αvβ5 and α5β1 integrin receptors [23,24]. In addition, structure-activity investigations using libraries of cyclic peptides and peptidomimetics have also provided several highly efficient low-molecular weights αvβ3, αvβ5 and α5β1 integrin antagonists [25,26]. αvβ3 integrin-selective cyclic peptide c(RGDfK) has found most widespread applications in targeted delivery of chemotherapeutics to tumor and tumor vasculatures [27,28,29,30].

## 3. Integrin-Selective Drug/Gene Delivery Platforms

Till date, a broad spectrum of anti-cancer therapeutics including small molecules, peptides, peptidomimetics, antibodies, small non-coding RNAs, etc. have been selectively delivered to tumor vasculatures via integrin receptors. Strategies employed span from direct covalent conjugation of drugs to integrin targeting ligands to utilizing integrin targeted nanoparticulate drug carriers for unloading the encapsulated cargo into tumor vasculatures. Important past and recent successes in the field are discussed below under different sub-headings based on the strategies adopted.

### 3.1. Integrin-Targeted Small Molecule Drug Conjugates

Over the past several years, a multitude of RGD peptides have been used in conjugation or in combination with cytotoxic drugs for selective homing to the tumor or tumor vasculature. In a landmark work, Arap and group [31] have conjugated Doxorubicin to a peptide sequence CDCRGDCFC which has been shown to selectively bind to αvβ3 and αvβ5 integrin receptors. The conjugate showed significant survival enhancement in a mouse model of human breast cancer xenograft when used at a nominal dose equivalent of Doxorubicin. Besides inducing significant damage to tumor tissue architecture, this conjugate was pronouncedly less toxic to liver and heart than free Doxorubicin.

An intelligent strategy to deliver chemotherapeutics is the use of prodrugs i.e., relatively non-cytotoxic forms of different drug molecules [32,33]. Prodrugs get converted into pharmacologically active species by metabolism or chemical cleavage after in vivo administration. Non-cytotoxic nature of pro-drugs enable them to be administered at significantly higher doses. RGD-based prodrugs have been synthesized by chemically conjugating RGD4C and Cilengitide with Doxsaliform [34]. The prodrugs are converted into an active metabolite of Doxorubicin with a half-life of 1 h under physiological conditions and penetrates the plasma membrane in MDA-MB-435 breast cancer cells. Besides Doxorubicin, anti-tumor efficacy of several other chemotherapeutic drugs has also been studied in conjugation with RGD based molecules. Chen and coworkers have developed a system containing Paclitaxel conjugated with dimeric RGD peptide i.e., E[c(RGDyK)]_2_ (RGD) [35] These Paclitaxel-RGD-conjugates inhibited cell proliferation via cell cycle arrest at G2/M-phase followed by apoptosis. Integrin specific accumulation of the conjugate was observed in vivo with significant tumor uptake at 2 h post-injection in mice bearing orthotopic MDA-MB-435 tumors.

αvβ3 integrin selective RGD peptides have been conjugated to anti-tubulin agent MonoMethyl Auristatin E (MMAE) or MonoMethyl Auristatin F (MMAF) via polyethylene glycol and albumin spacer [28]. MMAF-conjugates were reported to be more potent to induce killing of tumor cells than proliferating tumor endothelial cells.

In an attempt to increase the selectivity of Doxorubicin conjugates towards tumor vasculatures, Ryppa and group have reported on a matrix metalloproteinase-2/9 (MMP-2/MMP-9) cleavable conjugate of a divalent RGD peptidomimetic E-[c(RGDfK)_2_] [36]. Upon binding of the peptide conjugates to αvβ3 integrins, significant expression of MMP-2/9 in tumor vasculature was hypothesized to ensure drug release at the target site. Besides Doxorubicin and Paclitaxel, integrin-targeting peptides have been applied to conjugate Camptothecin [37,38].

The Vicent laboratory has conjugated polyglutamic acid to Paclitaxel to formulate a macromolecular drug of ~7 nm in diameter and ornamented with cyclic RGD [39]. Targeting αvβ3 integrin-expressing proliferating tumor endothelial cells with the conjugate blocked endothelial cell migration towards angiogenic inducers and showed tumor regression in an orthotopic mice tumor model. RGDfK-pHPMA-Docetaxel conjugate, ~3 nm in diameter, as reported by Ray and colleagues, was able to reduce growth of DU145 prostate cancer xenograft in a single dose [40]. Integrin-targeted poly(amidoamine) (PAMAM) dendrimer has been designed by Zhu and coworkers by conjugating 16.8 RGDyC units to one PAMAM unit in PEG-PAMAM-cys-aconityl-DOX (RGD-PPCD) [41,42]. Integrin-targeted conjugate showed increased tumor regression efficacy in B16 melanoma and C6 orthotopic glioma model in mice. To promote long-term drug retention, RGD-PPCD conjugated were encapsulated within implants composed of poly (dl-lactic-co-glycolic acid), poly (dl-lactic acid) and polyethylene glycol [43]. Enhanced tumor accumulation of Doxorubicin and better tumor regression efficiency were observed in C6 glioma mice tumor model for implants loaded with targeted conjugates compared to those for free drug and untargeted implants.

**Figure 3:**
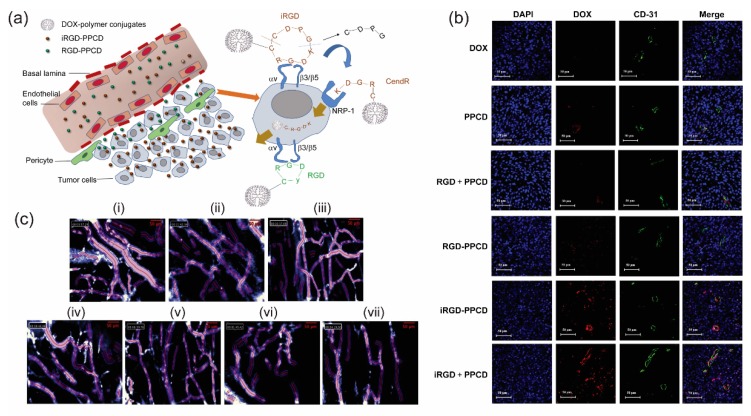
(**a**) Schematic elucidation of tumor penetrating characteristics of iRGD-PPCD and RGD-PPCD conjugates. PPCD denotes PEG-PAMAM-cis-aconityl-DOX. RGD-PPCD conjugates, upon binding to αv integrins, can access regions at the vicinity of tumor blood vessels, but possess limited capacity to penetrate tumor parenchyma. iRGD-PPCD conjugates, via neuropilin-1-dependent internalization, penetrates deeper into tumor parenchyma. (**b**) Assessment of tumor penetration of free DOX and conjugates in mice bearing intracranial C6 glioma, 12 h post intravenous administration. CD31 staining indicates blood vessel density with nuclei stained with DAPI. (**c**) Effects of individual treatment group on vasculature characteristics of subcutaneous C6 glioma tumor in mice. Vascular density and diameters are determined using Fibered confocal fluorescence microscopy. (i) Saline, (ii) DOX, (iii) PPCD, (iv) RGD + PPCD, (v) RGD-PPCD, (vi) iRGD-PPCD and (vii) iRGD + PPCD. Reproduced from reference [46] with permission from Elsevier.

Delivering therapeutics deep inside the tumor parenchyma against the enormous interstitial pressure remains a major challenge. To this end, Ruoslahti’s laboratory has identified a peptide motif, referred to as internalizing RGD (iRGD), R/KXXR/K, with ability of significant tumor tissue penetration [44,45]. Conjugation to a tumor-homing peptide iRGD (CRGDK/RGPD/EC) appreciably increases the sensitivity of tumor-imaging agent iron-oxide nanoworms as well as the potency of anticancer drug Abraxane. Mechanistically, tumor penetration is hypothesized to be a three-stage process where the entire peptide is first internalized through integrins αvβ3 or αvβ5 expressed on tumor endothelial cells. Exposure of the cryptic element R/KXXR/K by cell-surface proteases then allows its interaction to neuropilin-1 receptors and results in tumor penetration. Such penetration effect is distinctly different from EPR as it is energy-dependent as well as receptor-dependent process unlike the passive leakage from the tumor vessels in case of EPR. As Jiang laboratory reports, penetration efficacy into glioma spheroids for DOX-polymer conjugates (PPCD) carrying iRGD and conventional RGD were 144 and 115 µm, respectively [46]. iRGD-conjugated PPCD demonstrated enhanced permeability to tumor vasculature and significantly reduced vascular diameter than that for simple RGD-PPCD conjugates (Figure 3). Enhanced brain tumor accumulation of iRGD was reflected in increased survival of tumor bearing mice than mice treated with conventional RGD conjugates.

### 3.2. Integrin-Assisted Therapeutic Peptide and Protein-Conjugates

In addition to small-molecule drugs, integrin receptor-mediated targeting has been investigated to deliver therapeutic peptide and proteins to their specific site of action. Ellerby and coworkers have reported conjugation of CNGRC and RGD4C sequences to a pro-apoptotic peptide (KLAKLAK)_2_ [47]. The interesting design involved guiding the pro-apoptotic sequence to the tumor vasculature via the tumor-homing sequences. (KLAKLAK)_2_ domain triggered apoptosis only when internalized into tumor cells by mitochondrial membrane permeabilization and reduced chances of non-specific toxicity. Conjugation of RGD-4C to a naturally occurring anti-microbial peptide tachyplesin reduced colony-formation ability in TSU prostate cancer cells and induced tumor growth regression in syngenic mice models of prostate cancer and melanoma [48].

Tumor Necrosis Factor (TNF) is a cytokine that plays a dominant role in apoptosis, cell survival and immunity. Interestingly, αvβ3 integrin-specific ACDCRGDCFCG peptide conjugated to mouse TNF-α resulted in high binding-avidity [49]. Only a sub-nanogram level dose of the conjugate was sufficient to trigger significant tumor regression when the conjugate was combined with a chemotherapeutic drug melphalan. Conjugation of TRAIL (TNF related apoptosis inducing factor) with the ACDCRGDCFC peptide resulted in a dose-dependent binding in microvascular endothelial cells and exhibited high degree of apoptosis in αvβ3 and αvβ5-positive tumor cells [50]. In vivo anti-tumor efficacy of the conjugates was further enhanced in combination with irinotecan hydrochloride in mouse tumor xenograft models. As investigated by Jiang and coworkers, recombinant mutated human TNF-α (RGD-rmhTNF-α) showed ability to bind αvβ3 integrin in vitro, enhanced intratumoral uptake of Doxorubicin via increased permeability into tumor blood-vessels and synergistic anti-tumor efficacy with Doxorubicin in tumor xenograft models [51].

Besides TNF, endostatin and IL-12 have been conjugated to integrin-targeted peptides. IL-12, an interleukin produced by dendritic cells for stimulating growth and function of T cells, has adverse cytotoxicity which limits its therapeutic application. IL-12 when conjugated to RGD-4C, besides retaining the full potential of IL-12, significantly inhibited tumor progression in a neuroblastoma model as compared to native IL-12 [52]. Therapeutic potential of Endostatin, an endogenous inhibitor of angiogenesis has been enhanced by amino or carboxyl terminal RGD-modification of a point mutated endostatin [53]. The modification enhanced tumor localization and growth regression of ovarian and colon tumors in athymic mice.

### 3.3. Integrin-Targeted Lipid- and Polymer-Based Nanoparticles

Integrin-targeted nanomedicine is of special interest for successful delivery of drugs and genes to tumors and tumor vasculatures. Enhanced vascular leakiness added to the lack of proper lymphatic drainage in a properly vascularized solid tumor, popularly referred to as the Enhanced Permeability and Retention effect (EPR) [54], impart nanoparticles with a capacity of passive tumor targeting when their diameter falls in the range of 100–200 nm [55]. However, active-targeting based on selective binding of nanoparticles to certain membrane receptors overexpressed on tumors suggest an alternate strategy to account for patient heterogeneity and inherent complexity of tumor microenvironment. A combination of passive and active targeting could be advantageous to overcome tumor drug resistance and provide better therapeutic outcomes [9]. Size, shape and surface properties are of paramount importance when designing targeted nanoparticles. Cellular uptake of 100-nm nanoparticles was found to be 2.5-fold higher in comparison to particles of 1-μm diameter [56]. Similarly, surface-curvature is important to detect aggregation and receptor-binding ability [57]. A perfect integrin-targeted nanoparticulate system should reach its target at ease, get recognized by the receptor, bind to the integrins and deliver its payload to the tumor site with minimum drug loss to the healthy tissues. The current review highlights recent advances on integrin-assisted as well as lipid- and polymer-based nanomedicine.

#### 3.3.1. Small Molecule Drugs

Liposomes decorated with integrin-targeting ligands have been extensively studied for anti-angiogenic therapy [58]. These liposomes are often modified by introducing a polyethylene glycol (PEG) moiety to decipher stealth-like characteristics so the chances of non-specific interactions with the phagocytic cells can be reduced [59]. In vivo efficacy of several anticancer drugs has been enhanced using such sterically stabilized integrin-targeted liposomes as delivery vehicles. Xiong and coworkers have demonstrated the enhanced potency of Doxorubicin-containing liposomes with RGD motif tagged to the distal end of PEG coating. RGD motif containing liposomes facilitated Doxorubicin uptake in melanoma cells by integrin-mediated endocytosis [60]. RGD-modified liposomes exhibited higher in vitro cytotoxicity, enhanced circulation time and higher tumor uptake as compared to the unmodified liposomes. As a result, melanoma tumor regression was also significant for RGD-modified liposomes. Murphy’s group conjugated c(RGDfK) peptide to PEGylated liposomes to encapsulate Doxorubicin and the resulting nanoparticle showed a 15-fold decrease in tumor volume compared to the free drug without any significant weight loss, when administered systemically [30]. Similarly, c(RGDyK)-decorated PEGylated nanocarriers have been designed for pH-responsive delivery of Doxorubicin [61].

Recent work from Chaudhuri’s laboratory has demonstrated the implications of liposomes containing RGDK-lipopeptides to deliver the anti-cancer drug Curcumin to mouse tumor vasculatures. Mechanistically, the formulation was able to induce tumor growth regression via inhibition of Vascular Endothelial Growth Factor (VEGF)-induced STAT3 (Signal transducer and activator of transcription) phosphorylation [62]. With a purpose to increase circulation lifetime of the liposomes, RGDK-lipopeptide was PEGylated during the next stage of investigation. Application of liposomes functionalized with these PEGylated RGDK-lipopeptide for co-delivery of Doxorubicin and Curcumin to mouse tumor vasculature had resulted in 2–3-fold tumor regression compared to the formulations containing individual drugs [63].

Kokkoli and coworkers have reported a fibronectin-mimetic peptide PR_b that has a potential to specifically bind to integrin α5β1, thereby providing a tool to target tumor cells overexpressing integrin α5β1. Stealth liposomes containing PR_b peptide-amphiphile in the bilayer of the liposomes with encapsulated Doxorubicin were proved to be equally cytotoxic as free Doxorubicin [64]. Liposomes surface-modified with c(RGDyK) and loaded with Paclitaxel were able to reduce tumor volume by ~1.2-fold in a PC-3 tumor xenograft model when compared to the untargeted liposomes [65]. As shown by Dai’s group, mTOR inhibitor Rapamycin loaded inside PEG-PCL polymer micelles, when combined with Doxorubicin-loaded cyclic octapeptide liposomes targeting integrin α3, appreciable tumor growth reduction was observed in mice bearing triple negative breast cancer [66]. Liposomes containing c(RGDfC) peptide and loaded with antiangiogenic drug patupilone (EPO906) resulted in a potent antitumor effect in mice bearing neuroblastoma and RH30-rhabdomyosarcoma [67]. Selective recent advances on integrin-targeted nanoparticles for delivery of chemotherapeutics are highlighted in Table 1.

**Figure 4:**
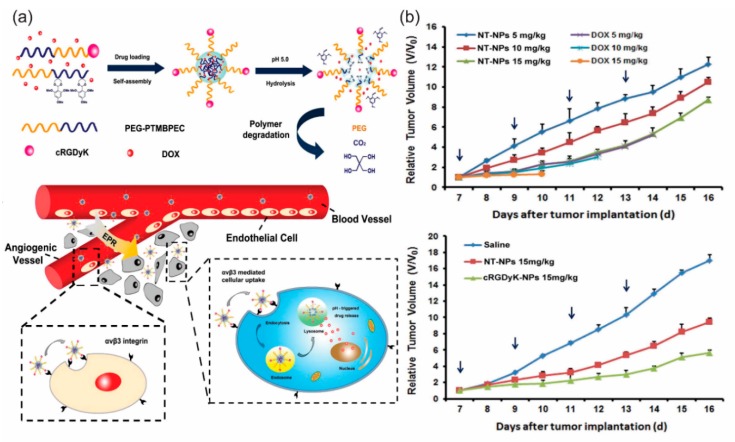
(**a**) Schematic diagram of self-assembly, disassembly and cellular internalization pathways of the c(RGDyK)-containing pH-sensitive nanoparticles into tumor and angiogenic endothelial cells. (**b**) Tumor growth inhibition in mice bearing B16 tumor with various doses of the nanoparticles. Tumor growth curve in mice treated with saline, NT-NPs and 10% c(RGDyK)-NP. Reproduced from reference [61] with permission from Elsevier.

Apart from liposomal delivery systems, numerous studies have been reported on the design of integrin-targeted polymeric nanoparticles. To name a few recent advances, cRGDyK-PEG-PLA micelles of ~35 nm in diameter were used by Zhan and coworkers to encapsulate Paclitaxel [75]. Targeted micelles showed a seven-fold decrease in tumor volume compared to control in a U87MG glioblastoma mouse model and increased mean survival of mice. Similar micelles have also been used to encapsulate Docetaxel [74] and curcumin [78]. PEO-PCL micelles decorated with RGD4C having Doxorubicin conjugated to the micellar core via acid-sensitive hydrazone linkage exhibited enhanced tumor accumulation in a DOX-sensitive LCC6^WT^ tumor model [76]. Similar micelles with DOX conjugated via a stable amide linkage showed higher efficacy in a DOX-resistant LCC6^MDR^ model. As shown by Kataoka’s group, PEG-PGA micelles incorporating platinum-based anticancer drug (1,2-diaminocylohexane) platinum (II), demonstrated fast accumulation in the tumor tissue in U87MG orthotopic glioblastoma model when their surface was decorated with 20 mol % cRGDfK as compared to the untargeted cRAD micelles [79]. The targeted micelles were also equally effective in inhibiting lymph node metastasis in a syngeneic melanoma model [80]. cRGDyK was used by Qiu and coworkers to decorate polymeric nanoparticles composed of a block-copolymer poly(ethylene glycol)-poly(2,4,6-trimethoxy benzylidene pentaerythritol carbonate). These pH-sensitive nanoparticles efficiently incorporated Doxorubicin and showed significant tumor regression efficacy [61] (Figure 4). An interesting development in the polymeric nanoparticle domain is polymersome, a vesicle generated via self-assembly of block copolymers. iRGD-conjugated pH sensitive polymersomes of poly (oligoethylene glycol methacrylate)-poly(2-(diisopropylamino) ethyl methacrylate) has been successfully used to encapsulate Paclitaxel and the resulting vesicle has shown significant anti-tumor effect in peritoneal tumors from gastric (MKN-45P) or colon (CT26) origin [81]. Usual limitations of polymeric nanoparticles such as non-specific interactions with blood components due to excess positive charge can be controlled by designing charge-reversal polymers. These are usually acid-labile amidized cationic polymers which stay inert in the circulation but can be activated in the tumor target sites via pH alteration [82]. To address circulation-instability of cargo before it could reach the tumor target, covalent conjugation is an interesting alternative to simple encapsulation. Stimuli-responsive polymeric nanoparticles can ensure sustained protection of payload until internalization into the target cells where the encapsulated drug or protein could be released in response to certain triggers like light, pH, redox conditions, temperature, etc. [83,84].

Each class of integrin-targeted nanoparticulate delivery system has its unique advantages and shortcomings. Liposomes have distinct advantages of encapsulating hydrophilic drugs at their aqueous compartments vs hydrophobic drugs at the lipid bilayers. A size range of 50–1000 nm for liposomes which is appreciably larger than 10–100 nm size of micelles might provide the advantage of higher drug loading which is also evident from Table 1. However, liposomes have the limitation of fast elimination via the reticuloendothelial system [85]. Polymeric micelles may have limited stability, slow extravasation and liver toxicity due to slower clearance despite having the advantages of conjugating multiple drug molecules via the multiplicity of polymer functional groups [86]. Interestingly, small-sized protein-loaded PEG-lipid micelles have demonstrated more efficient targeting of Lewis Lung Carcinoma than long-circulating large liposomes [87]. Comparative studies on the application of liposomes, micelles and polymeric nanoparticles side-by-side for anti-angiogenic therapy are missing in the literature which may shed light on new design concepts. Lipid-polymer hybrid nanoparticles which include complimentary characteristics from polymer and lipid-based nanoparticles could be interesting candidates for future investigations [88,89].

#### 3.3.2. Nucleic Acids

Integrin-assisted and nanoparticle-mediated delivery of therapeutic nucleic acids is a promising alternative to viral vectors as a strategy to target genetic mutations observed in cancer (Table 2). Immunogenicity and a lack of specificity of viral vehicles have been addressed via development of non-viral targeted nanoparticles [90]. Cheresh’s laboratory has reported the use of a cationic lipid-based nanoparticle containing an αvβ3 integrin targeting ligand to selectively deliver a mutant Raf gene to angiogenic blood vessels in tumor-bearing mice [91]. Apoptosis of tumor endothelium ultimately resulted in tumor cell apoptosis and significant regression of primary and metastatic tumor burden. Recently, Chaudhuri and coworkers have demonstrated that once an RGD-lipopeptide is modified with a lysine residue at the C-terminus, liposomes containing these non-cyclic conformationally unstrained tetrapeptide are able to target genes to mouse tumor vasculatures via α5β1 integrins [92]. A similar observation from the same group reported that cationic amphiphiles containing the RGDGWK hexapeptide sequence can deliver genes to the cultured cells preferably via α5β1 integrins. A significant tumor growth inhibition was observed when an electrostatic complex of anti-cancer gene *p53* and the liposomes was intravenously administered into mice bearing aggressive B16F10 tumors [93]. Integrin-targeted stealth liposomes were as equally effective as siRNA carrier in vivo. Liposomes with diameters of 160–180 nm functionalized with RGDK-PEG lipopeptide carrying encapsulated siRNA targeted to the cell cycle regulator CDC20 were able to target tumor and tumor endothelial cells via integrin α5β1 when tested in vitro. Significant growth inhibition of mice melanoma tumor was observed upon systemic injection of these carriers. Inhibitory effect was accompanied by an appreciable degree of apoptosis in tumor and endothelial cells and reduction in tumor blood vessel density [94]. A cell-penetrating peptide-decorated liposomal platform has also been reported by Chaudhuri’s laboratory and has been explored to deliver anti-angiogenic siRNA targeted against VEGF in mice bearing melanoma tumor [95].

Wang et al. have developed an integrin-targeted siRNA delivery system based on a multifunctional lipid carrier abbreviated as EHCO. The study was aimed at delivering anti-HIF-1a siRNA with the intention to knockdown the expression of HIF-1a, a key transcription factor responsible for the survival of cancer cells in hypoxic environments [96]. c(RGDfK) was conjugated to the lipoplex surface through a PEG moiety by a sulfhydryl-malimide coupling reaction. This RGD-based delivery system had shown significant tumor regression in athymic mice bearing human glioma U87 xenografts. Another example of integrin-mediated siRNA delivery has been reported by Schiffelers et al. where self-assembling nanoparticles containing PEGylated polyethyleneimine with RGD motif present at the distal end of PEG moiety have been used to deliver siRNA targeted against Vascular Endothelial Growth Factor Receptor-2 VEGFR2 [97]. The nanoparticles selectively accumulated in tumors and significantly reduced expression of the *VEGFR2* gene as well as tumor angiogenesis, leading to tumor regression. Selective recent advances on integrin-targeted nanoparticles for nucleic acid delivery are highlighted in Table 2.

Positively charged RGD-lipid-Protamine nanoparticles with diameters of 222 nm were used to deliver siRNA targeted against PAX3-FOXO1 (P3F), a fusion transcript expressed in alveolar rhabdomyosarcoma (ARMS) [99]. Statistically significant tumor growth delay was observed in a xenograft ARMS model. Integrin targeted P123 Pluronic block copolymers were used to compose nanoparticles of ~ 20 nm diameter tagged with c(RGDfK) with an aim to encapsulate AP-2α (activating protein 2α) protein expression plasmid [104]. AP-2α plays an important role to inhibit anti-apoptotic Bcl2 and upregulate apoptotic Bax/Cytochrome/Apaf1/caspase-9 network. Four-fold reduction in tumor size compared to untargeted conjugate was observed in a primary gastric tumor animal model. Systemically injected cRGD-modified polyion complex-assembled gold nanoparticles could target HeLa tumor and have shown enhanced gene silencing ability in the tumor [108].

#### 3.3.3. Combination Delivery of Nucleic Acids and Drugs

**Figure 5:**
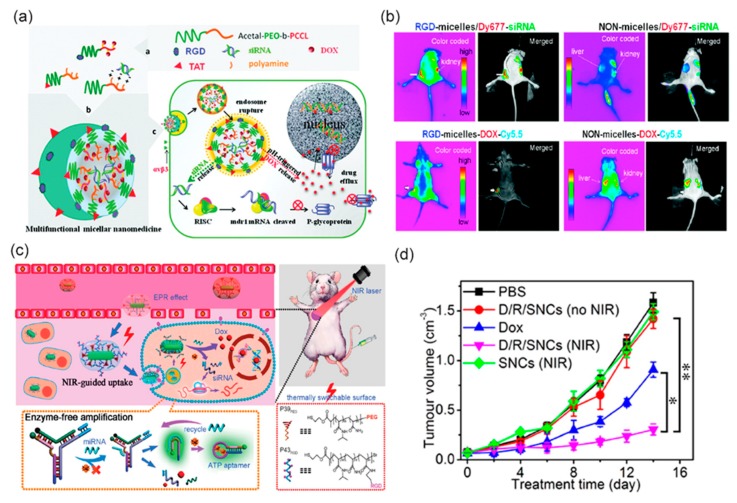
(**a**) Schematic diagram of multifunctional micellar nanoparticles for targeted co-delivery of siRNA against P-gp and DOX designed to overcome multidrug resistance. Drugs are encapsulated at the micellar core and the shell of the micelles are modified with RGD or TAT peptides. (**b**) In vivo near-infrared fluorescent imaging of athymic nude mice bearing MDA-MB-435/LCC6MDR1-resistant tumors upon intravenous administration with RGD- and NON-micelles-DOX-Cy5.5 and RGD- and NON-micelles/Dy677-siRNA. Reprinted (adapted) with permission from reference [106], Copyright © 2011 American Chemical Society. (**c**) Schematic illustration of smart nanocarriers (SNC) capable of NIR-mediated siRNA and DOX co-delivery with endogenous miRNA and ATP as the controllers of intracellular cargo release. (**d**) Tumor growth inhibition efficacy of the SNCs in mice bearing subcutaneous HeLa tumors. * *p* < 0.05, ** *p* < 0.01 (two tailed Student’s t-test). Reprinted (adapted) with permission from reference [109], Copyright © 2011 American Chemical Society.

Non-specific distribution of chemotherapeutic drugs and development of multi-drug resistance often impairs success of chemotherapy. Resistance to common drugs like anthracyclines and taxanes is commonly associated to overexpression of drug-transporter protein P-gp encoded by MDR1 gene in tumor cells. Prior inhibition of P-gp via targeted siRNA delivery is an attractive strategy with potential to increase the level of drug accumulation into tumor. To this end, Xiong and group designed a micellar system from poly (ethylene oxide)-block poly (εcaprolactone) (PEO-b-PCL) block copolymers where PCL block has been functionalized with polyamines for chemically conjugating siRNA or Doxorubicin via hydrazone linkages (Figure 5a,b). The system was further modified with RGD4C motif for αvβ3 integrin-mediated tumor targeting and tagged with cell-penetrating peptide Tat for increased membrane fusion. The micelles were able to release Doxorubicin in a pH dependent mechanism and demonstrated significant cellular uptake, improved dox penetration in vitro and tumor specific accumulation in multi-drug resistant MDA-MB-435 human tumor models [106].

In a different strategy used by Jiang and coworkers, RGD-PEG-DSPE containing cationic liposomes carrying MDR1 siRNA has been shown to be preferentially accumulated in drug-resistant MCF7/A tumors and reversed the drug resistance by downregulating P-gp expression level in the tumor cells [101]. Four doses of siRNA-liposome complex were administered via the tail vein in BALB/c mice bearing MCF7/A tumors, following the administration of Doxorubicin encapsulated in the same targeting liposomes. This sequential administration of liposomal siRNA and drug significantly reduced the growth of drug-resistant tumors compared to the regression in case of liposomal drug alone. Overall, such a combination strategy of chemotherapeutic drugs and siRNA could be a promising modality for treating drug-resistant tumors.

Positively charged iRGD-TPGS/Pluronic P85-PEI nanoparticles with diameters of 141–160 nm have been used by Shen and coworkers to co-deliver Paclitaxel and short hairpin RNA targeted against Survivin, an apoptosis inhibitor protein [102]. ~80% downregulation of Survivin expression was observed in the tumor tissue which resulted in 2–3-fold tumor volume regression compared to the untargeted control group. Near-infrared guided smart nanocarriers composed of a stimuli-responsive DNA Y-motif, two temperature-sensitive polymer and gold nanorods were constructed by Zhang and colleagues to co-deliver Doxorubicin and PLK1 siRNA [109]. The interesting design feature allowed reversible photothermal interconversion between PEG and RGD decoration on the surface while drug release was mediated via endogenous miRNA and ATP as stimuli (Figure 5c,d).

## 4. Is RGD-Mediated Targeting Good Enough?

The RGD motif has been one of the most explored ligands for antiangiogenic therapy. RGD-mediated therapy has its distinct advantages since the target integrins, like αvβ3, are expressed on angiogenic tumor endothelial cells in addition to the tumor cells in numerous cancers [12,110], while not on the quiescent vasculatures. A targeting ligand may not play a role in the overall tumor accumulation of the nanoparticles if the target is not adequately expressed on the tumor vasculature. In such case, targeted and non-targeted particle often show similar tumoral uptake via EPR effect [111,112,113]. Transferrin-targeted nanoparticles formed with cyclodextrin-containing polycations and siRNA showed similar tumor localization compared to the non-targeted control nanoparticle as determined via PET [114]. Similarly, folate-targeting did not alter tumor accumulation of liposomes in mouse M109 and human KB carcinomas, and mouse J6456 lymphoma [115]. Integrin-targeting can be distinguished from folate- and transferrin-mediated targeting since the expression of these two widely used receptors are limited to cancer cells only [8]. RGD-decorated nanoparticles can achieve dual targeting via initial binding to the integrins in the tumor blood vessels followed by binding to the integrins of tumor cells after extravasation. On the other hand, folate supplied through food intake possibly competes with receptor-binding of folate-targeted nanoparticles, which may ultimately reduce concentration of intracellularly delivered drug [116].

Even though RGD is the most sought-after ligand for integrin-mediated angiogenesis, a few other integrin-binding peptides have also been explored for similar purposes. The laminin-derived peptide sequence SIKVAV known to bind integrins α3β1 and α6β1 has been explored to design gold nanoparticles to enhance target-specific uptake [117]. Similar sequences when used to decorate a polymer-coated virus allowed tumor-specific uptake of the modified virus via α6 integrins [118]. Apart from integrin-binding peptides, those targeting a handful of other pro-angiogenic receptors have been exploited to design nanoparticles. Peptide KPQPRPLS targeting Vascular Endothelial Growth Factor Receptor-1 and peptide KATWLPPR targeting Neuropilin-1 have been used to functionalize gold nanoparticles with an aim to alter the balance between Pro- and anti-angiogenic factors [119]. Surface modification of nanoparticles with laminin-binding peptide YIGSR has been shown to improve targeting to metastatic melanoma [120]. Heparan sulfate receptors expressed on angiogenic endothelial cells have been selectively targeted using CGKRK peptide-modified PEG-co-PCL nanoparticles in a U87MG brain tumor model [121].

Multivalent targeting through simultaneous binding of multiple receptors may provide an interesting avenue to increase tumor accumulation of delivered drugs. Poly-(lactide-co-glycolide) nanoparticles dually functionalized with RGD and transferrin showed increased accumulation in retinal cells and highly reduced laser-induced choroidal neovascularization in rats [122]. Similarly, αvβ3-integrin- and galectin-1-targeted paramagnetic liposomes showed synergistically elevated uptake in the treatment of angiogenesis [123]. Given the heterogeneity of receptor expression across tumors, developing screening techniques where nanoparticles of varying receptor-targeting functionality and biophysical characters (shape, size, charge, etc) can be rationally identified for specificity, affinity and avidity of binding.

## 5. Future Perspectives

The integrin receptor has been a star player in targeted drug delivery as clearly evidenced in recent literature. Integrin-targeted nanoparticles with diameters of 100–200 nm offer dual advantages of active-targeting and passive EPR-mediated effect which help enhance therapeutic efficacy by reducing drug dosage and often imparting selective tumor targeting ability. Unfortunately, studies aimed at comparing efficacies of distinct delivery systems are still missing. A wide variety of polymer and lipid-based systems have been studied to deliver a plethora of drugs and nucleic acids, often in different tumor models and with a wide range of dosage, making it difficult to determine their comparative potency. Most studies have only looked at the relative capacity of targeted vs non-targeted vehicles. Efficiency of delivery as determined via percentage of accumulated drug dose at the tumor site [124] (vs. vital organs) is largely missing in literature. In addition, anti-tumor efficacy of the nanoparticles has been evaluated solely based on tumor volume shrinkage rather than ensuring a complete remission. Such temporary anti-angiogenic effect has been shown [125] to induce invasion and metastasis necessitating development of targeted therapy for intervening on both localized early stage and metastatic late stage tumors [126].

Despite promising preclinical results, only two integrin-targeted systems are being investigated in clinical trials [127] (ClinicalTrials.gov identifier NCT02106598) which seemingly suggests shortcoming of carrier design and a lack of effective preclinical models able to closely mimic clinical tumor microenvironment. Complexity of heterogeneous tumor tissue composed of cancer stem cells, blood vessels, immune cells and extracellular matrix can be accurately studied by recently developed organ-on-a-chip technology [128]. Cutting-edge engineering of chips to recapitulate interactions between tumor and stroma, tumor vs vasculature and tumor vs extracellular matrix components can aid in more accurate prescreening of the targeted nanoparticles than in conventional 2D cell culture. High degree of reproducibility in the production of nanoparticles achieved via microfluidic technology is helpful for large-scale production [129,130]. Preclinical investigations should elucidate in-depth evaluation of immunogenic effect and non-specific toxicity of the vehicle in addition to tissue distribution and pharmacokinetic profiles. There is room for improvement in design strategies to generate integrin ligands with even higher specificity. Detailed structure–activity relationships with due consideration to zeta potential, hydrodynamic diameter, flexibility of ligand-display, multi-valency and spatial segregation of ligands, shape of the vehicle [55], drug-encapsulation and release-kinetics are needed to be investigated for proper optimization of the vehicle [131,132]. In-depth research is needed to address the effects of integrin internalization, recycling and saturation to control the delivered drug. Reproducibility of preclinical data and evaluation in patient-derived tumor models are the key steps to consider. Heterogeneity of receptor expression across patients and even across different regions of the same tumor certainly poses challenge towards active-targeting. A more refined understanding of angiogenic process through Big data produced from genomics, transcriptomics and proteomics can aid in developing precision nanotherapeutics [133]. Prior determination of integrin expression in biopsy samples from individual patients might provide a more defined clinical strategy. A close collaboration between bench scientists and clinicians could help overcome the large gap that still exists between preclinical and clinical outcomes of anti-angiogenic therapy.

## Figures and Tables

**Table 1:** A non-exhaustive list of integrin receptor-targeted small molecule drug delivery systems.

Vehicle Type	Integrin-Targeting Sequence	Drug	Loading/ Encapsulation	Mice Tumor Model	References
Liposome	cRGDf	Doxorubicin	80–150 µg per µmol of lipid/ND	C26 carcinoma	[29]
	RGDm	Doxorubicin	Lipid/drug 15:1 (*w*/*w*)/ND	B16 melanoma	[60]
	RGD	Paclitaxel	ND/90%	SKOV-3 ovarian cancer	[68]
	c(RGDyK)	Paclitaxel	5% of the SPC + cholesterol weight/84%	PC-3 prostate cancer	[65]
	cRGDfC	EPO906	ND/˃95%	Kelly neuroblastoma, RH-30 rhabdomyosarcoma	[67]
	Ac-CRGDS	Gemcitabine/Pirfenidone	50–2000 µg/50% (Gemcitabine), 10–100 µg (Pirfenidone)/98% (Pirfenidone)	PSCs/Panc-1 pancreatic cancer	[69]
	RGDK	Curcumin	Lipid/drug 15:1 (*w*/*w*)/85–90%	B16F10 melanoma	[62]
	PEG-RGDK	Curcumin/ Doxorubicin	Lipid/drug 10:1 (*w*/*w*)/74% (Curcumin) & 100% (Doxorubicin)	B16F10 melanoma	[63]
Polymeric nanoparticle	cRGDf	MMAE/F	ND/ ND	C26 carcinoma	[70]
	iRGD	Paclitaxel	5 mg drug per 20 mg of copolymer/˃90%	H22 hepatic tumor	[71]
	c(RGDyK)	Doxorubicin	10%/60%	B16 melanoma	[61]
	c(RGDfK)	Pt (IV)	30–50% of the polymer (*w*/*w*)/3%	MCF7MFP1 breast cancer	[72]
Polymeric micelle	RGD4C	Paclitaxel	1.25 mg drug per 2.5 mg of polymer/ ND	MDA-MB-435 breast tumor	[73]
	c(RGDyK)	Docetaxel	5 mg of drug per 10 mg of PLA-PEG/90%	U87MG glioblastoma	[74]
	c(RGDyK)	Paclitaxel	10–50% (*w*/*w* to polymer)/98–63%	U87MG glioblastoma	[75]
	RGD4C	Doxorubicin	ND/ND	MDA-435 LCC6 derived tumors	[76]
Polymer conjugate	iRGD	Doxorubicin	NA	C6 glioma	[46]
	RGDfK	Aminohexyl-geldanamycin	20–27%	DU145 prostate cancer	[77]

ND: Not discussed; NA: Not applicable.

**Table 2:** A non-exhaustive list of integrin-receptor mediated nucleic acid delivery systems.

Vehicle	Integrin-Targeting Sequence	Nucleic Acid +/− Drug	Nucleic Acid Encapsulation	Mice Tumor Model	References
Liposome	RGDK	p-CMV-p53	ND	B16F1 melanoma	[92]
	RGDGWK	p-CMV-p53	ND	B16F10 melanoma	[93]
	PEG-RGDK	CDC20 siRNA	88–90%	B16F10 melanoma	[94]
	c(RGDfK)	VEGFR2 siRNA	˃90%	OS-RC-2-kidney carcinoma	[98]
	RGD	PAX3-FOXO1 siRNA/ protamine	ND	Rh30 alveolar rhabdomyosarcoma	[99]
	mAb E7P6	Integrin β6-siRNA	85–87%	HT-29 colon cancer	[100]
Polymeric nanoparticle	RGD-PEG-DSPE	MDR1siRNA/Doxorubicin	ND	MCF7/A tumor	[101]
	iRGD	Survivin shRNA/ Paclitaxel	94%	A549/T lung cancer	[102]
	GRGDSPK	STAT1 mRNA	75%	Collagen-induced arthritis	[103]
	c(RGDfK)	pCMV6-AP-2α	67%	MGC803 gastric tumor	[104]
	RGD4C	MCL-1 siRNA	ND	MDA-MB-435 breast cancer	[105]
Polymeric micelle	RGD4C	P-gp siRNA/ Doxorubicin	ND	MDA-MB-435 Breast tumor	[106]
Polyplex	c(ACDCRGDCFC)	VEGFR1 siRNA	ND	CT-26 colon adenocarcinoma	[107]

ND: Not discussed.
